# Membranoproliferative glomerulonephritis with essential cryoglobulinemia

**DOI:** 10.4103/0971-4065.42347

**Published:** 2008-04

**Authors:** S. Satish, R. Rajesh, K. George, E. M. Elango, V. N. Unni

**Affiliations:** Department of Nephrology, Amrita Institute of Medical Sciences, Cochin, India; 1 Department of Molecular Biology, Amrita Institute of Medical Sciences, Cochin, India

**Keywords:** Cryoglobulinemia, membranoproliferative glomerulonephritis

## Abstract

Cryoglobulinemia is an uncommon cause of renal disease and often occurs in patients with hepatitis C virus (HCV) infection. We report a case of membranoproliferative glomerulonephritis in a patient with cryoglobulinemia, which was not associated with HCV infection or any identifiable etiology.

Cryoglobulins are monoclonal or polyclonal immunoglobulins which precipitate as serum is cooled below core body temperatures. Although the term cryoglobulinemia refers to the presence of cryoglobulins in serum, it is often used to denote a systemic inflammatory syndrome involving small to medium sized blood vessels due to cryoglobulin containing immune complexes. Renal involvement is common and denotes poor long term prognosis.

## Case Report

A 34-year-old man was admitted for evaluation of worsening pedal edema. He was apparently healthy until four years back, when he developed edema of both feet and nonpruritic macular skin rash involving both lower limbs. Subsequently, he was detected to have hypertension, proteinuria and mild renal failure (6 months ago). He had no history of arthralgia, Raynaud's phenomenon, paraesthesia or gastrointestinal hemorrhage.

Six weeks prior to admission in our centre, he developed weakness of left upper and lower limbs with slurring of speech and was hospitalized elsewhere. A computerized tomographic scan of head had revealed an infarct in the right middle cerebral artery territory and he was treated with aspirin, statins, and antihypertensives. He made a complete recovery but was detected to have renal failure and was referred to us for further evaluation.

He weighed 74 kg and had a height of 174 cm. He had pallor and bilateral pitting pedal edema. There was no skin rash or lymphadenopathy. Blood pressure was 140/90 mmHg. Examination of cardiovascular system, respiratory system, and abdomen was normal. There were no neurological deficits.

**Fig. 1:**
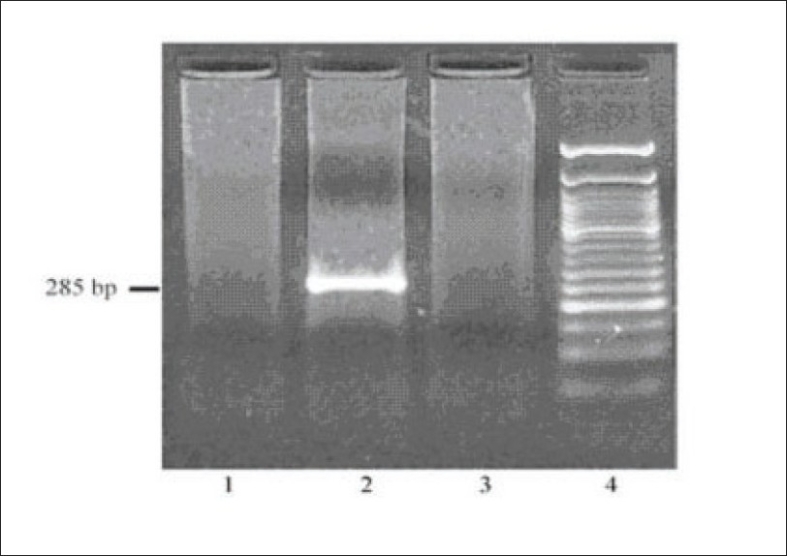
RT-PCR for HCV RNA in the cryoprecipitate. Lane 1 shows negative control. Lane 2 shows a positive control, lane 3 the test sample and lane 4, the 50 base pair (bp) DNA ladder. Absence of 285 bp amplified PCR product in the test lane (lane 3) indicates that the sample is negative for HCV

**Fig. 2:**
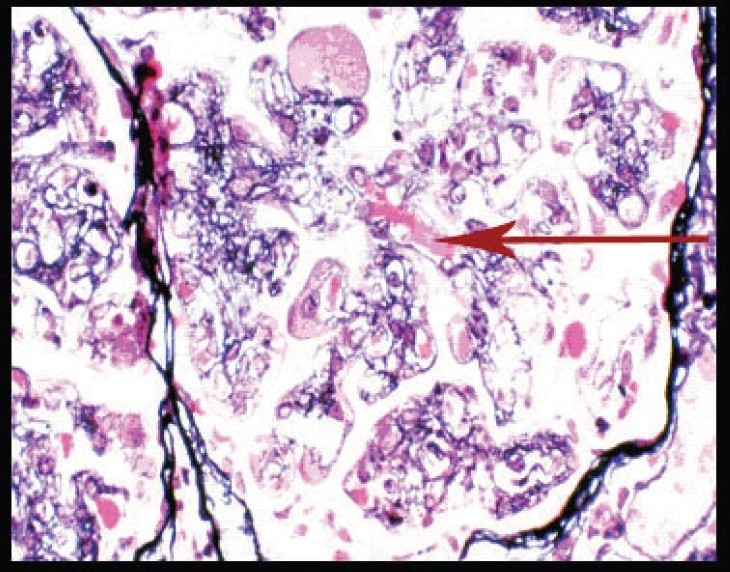
Silver methenamine stain showing basement membrane splitting, interposition of mesangium, and intracapillary hyaline like deposits (arrow) (magnification ×400)

Investigations revealed hemoglobin of 8.79 gm/dL, platelet count of 112000/mm^3^, total WBC count of 7960/mm^3^ - with a normal differential count, ESR of 40 mm at end of first hour. Urinalysis showed protein ++++, 6-8 RBCs/HPF; and 24 hour urine protein was 4420 mg. Blood urea was 72 mg/dL and serum creatinine was 3.1 mg/dL with normal serum levels of electrolytes. Liver function tests, PT and aPTT were normal. Serum albumin was 3.6 gm%, serum globulin 3.8 g/dL and serum cholesterol 240 mg/dL. Serum protein electrophoresis was normal. Blood and urine cultures were sterile. HBsAg, antihepatitis C virus (HCV) and HIV antibodies were negative VDRL, cANCA, pANCA, antiphospholipid antibodies, ANA, antids DNA were negative. Serum homocysteine [12 µmol/L] was normal and serum complement levels were reduced (C_3_:70 mg/dL, C_4_:18 mg/dL). Serum cryoglobulin assay was positive. On PAGE electrophoresis, the mobility was suggestive of mixed cryoglobulins. HCV RNA was negative in serum [polymerase chain reaction (PCR)]. Qualitative analysis for HCV RNA [by reverse transcriptase-PCR (RT-PCR)] in the cryoprecipitate was negative [Fig. 1]. Doppler study of extra cranial carotid and vertebral vessels was normal. Ultra sonogram of abdomen, ECG, and echocardiogram were normal. Bone marrow examination was normal. X-rays of chest, skull spine, and pelvis were normal. Kidney biopsy showed lobular accentuation of glomeruli with mesangial proliferation, mild endocapillary proliferation, and double contour of glomerular basement membrane on light microscopy. Intracapillary eosinophilic PAS positive hyaline deposits were noted [Fig. 2]. Interstitium showed mononuclear infiltrates. Immunofluorescence showed mesangial and capillary granular positivity for C_3_ IgG and IgM.

## Discussion

Cryoglobulins are immunoglobulins that undergo reversible precipitation at low temperatures. They were first described by Wintrobe and Buell in 1933.^1^ They are classified into three types.^2^ Type I cryoglobulin is a single monoclonal immunoglobulin associated with plasma cell dyscrasias. Type II cryoglobulins are mixed cryoglobulins consisting of a monoclonal antiglobulin (usually IgM) plus polyclonal IgGs. Type III cryoglobulins are mixed polyclonal immunoglobulins. Type II and type III are defined as mixed cryoglobulinemias. The majority of mixed cryoglobulinemias (MC) have been detected in patients with connective tissue disorders, lymphoproliferative disorders, noninfectious hepatobiliary diseases or infectious diseases, with nearly 30% previously considered as “essential”. With the advent of serological and antigenic markers for HCV, it was clear that the majority of these essential cryoglobulinemias are linked to HCV.

Testing for serum cryoglobulins may be carried out in two stages.^3^ The first stage involves an initial screening. Values of screening are expressed as cryocrit, which is the volume percentage of the precipitate compared to the total volume of serum. The second stage requires more detailed characterization and typing of the cryoglobulins.

Once it is decided to test for cryoglobulins, it is critical to collect the serum properly. Common errors include loss of cryoglobulins due to failure in properly separating serum from whole blood, loss of cryoprecipitate due to refrigeration before centrifugation, and an inadequate volume of serum for testing of cryoglobulins which may be present at low levels.^3^

It is recommended that 10-20 mL of blood be collected in a tube or syringe prewarmed to 37°C and the sample allowed to clot at this temperature for 30-60 min prior to separation. Since cryoglobulin levels may vary from 50-100 µg/mL to 5-10 mg/mL or higher, the volume of serum necessary for complete characterization may vary widely. Blood samples may be kept in a water bath of 37°C or placed in a thermos filled with sand kept at this temperature, prior to transfer to the laboratory. Serum is separated from the clot by centrifuging warm for 10 min at 2500 rpm. Following centrifugation, the serum is checked for lipemia, which may complicate visual inspection of the sample later, for cryoprecipitation. Serum processed as above is kept at a temperature of 4°C and tested for cryoprecipitation by daily inspection for 7 days. A parallel aliquot may be kept at 37°C for comparison. Following detection of a precipitate, some effort has to be taken to show that it is soluble at 37°C, preferably by rewarming a small portion.^3^

Methods for typing the cryoglobulins include immunofixation, immunoblotting, two-dimensional gel electrophoresis, and capillary zone elctrophoresis. Currently, immunofixation is the method of choice for accurate assessment of clonality in serum and for typing the cryoglobulins. It is important that these tests are done with the serum kept at 37°C as they may be influenced by artifacts arising from *ex vivo* precipitation of the cryoglobulins, if the serum gets cooler. HCV infection may be detected by immunologic testing of antibodies or by detection of viral RNA. HCV testing in serum of patients suspected or proven to have cryoglobulinemia may be quite unreliable, largely due to loss of antibody or HCV RNA during processing. Occasional instances have been recorded in which PCR quantification was felt to be negative, until measurements were carried out on the cryoprecipitate.^3^

Typical clinical presentations and reported frequencies of specific manifestations of cryoglobulinemia are as follows:^1^ cutaneous manifestations (55-100%), articular manifestations (35-72%), renal involvement (8-57%), Raynaud's phenomenon (28-50%), neurological symptoms (10-30%), abdominal pain (2-22%), acrocyanosis (9%), hemorrhage (7%), and arterial thrombosis (1%). Renal involvement occurs mostly in the MC with a higher prevalence in type II MC. Generally, systemic symptoms antedate renal involvement. Renal disease occurs at presentation in less than 25% of patients, but develops in about 50% over time. More than a half has proteinuria and/or hematuria only. Nephrotic syndrome is diagnosed in 20% of patients and acute nephritic syndrome in 20%. The majority have an indolent course. Renal histology shows varying degrees of endocapillary proliferation in almost all patients with MC. In two-thirds, the proliferative changes together with the thickening/reduplication of GBM assume the picture of MPGN. On immunofluorescence, deposition of immunoglobulin and complement are always present. Cryoglobulinemic MPGN may appear histologically identical to MPGN type I by light, immunoflourescence and electron microscopy.^4^ However, a more pronounced mononuclear infiltration and visualization of the cryoprecipitates as intracapillary hyaline like deposits are major differences. Immunofluorescence shows subendothelial and mesangial deposits of IgM, IgG, C_3_ and frequently C_1q_.

Patients with only systemic signs of MC may have an indolent course for years, with alternating periods of exacerbations and spontaneous quiescence. The prognosis of patients with MC is worsened by renal disease.^4^

Acute nephritic and/or severe vasculitic flare-ups may be treated with high-dose intravenous steroid pulses followed by a short-term course of oral prednisolone and cytotoxic drugs.^4^ Life-threatening refractory symptoms may be considered an indication for plasmapheresis or cryofiltration apheresis.^4^ Given its natural history, prolonged immunosuppression does not appear to be justified in patients with MC. With recognition of the association of MC with HCV infection, the emphasis of treatment of both renal and systemic manifestations has shifted to antiviral therapy. Combination regimens of interferon α and ribavirin for at least 6 months has been reported to have a sustained virologic response (defined as undetectable serum HCV RNA levels 6 months after completion of treatment) in 40-45% of patients.^5^ In patients who progress to end stage renal disease, dialysis and transplantation have been used; recurrences in the graft have been reported.

There is a small but significant proportion (10%) of MC that are not associated with any known etiology, as in this patient. These can be considered truly “essential”. A search for as yet unknown etiologic factors may be required in these patients. As this patient did not have any evidence of HCV infection and had a fairly stable renal function, it was decided to manage him conservatively. He has been doing fairly well with a stable serum creatinine and is on our regular follow-up for nearly 2 years.

Cryoglobulinemia is uncommonly encountered in Nephrology practice. The screening and characterization of cyroglobulins can be technically demanding. Although a workup for the various causes of cryoglobulinemia needs to be done, it must be remembered that in a fraction of patients, it may be idiopathic (essential).
